# Biomarkers over Time: From Visual Contrast Sensitivity to Transcriptomics in Differentiating Chronic Inflammatory Response Syndrome and Myalgic Encephalomyelitis/Chronic Fatigue Syndrome

**DOI:** 10.3390/ijms26157284

**Published:** 2025-07-28

**Authors:** Ming Dooley

**Affiliations:** Holistic Resonance Center, 2525 Camino Del Rio S #130, San Diego, CA 92108, USA; ming@holisticresonancecenter.com

**Keywords:** chronic inflammatory response syndrome (CIRS), Myalgic Encephalomyelitis/Chronic Fatigue Syndrome (ME/CFS), biomarkers, visual contrast sensitivity (VCS), transcriptomics, immune dysregulation, neuroendocrine markers, water-damaged buildings (WDBs), mold exposure, environmental biotoxins

## Abstract

Chronic inflammatory response syndrome (CIRS) and Myalgic Encephalomyelitis/Chronic Fatigue Syndrome (ME/CFS) are debilitating multisystem illnesses that share overlapping symptoms and molecular patterns, including immune dysregulation, mitochondrial impairment, and vascular dysfunction. This review provides a chronological synthesis of biomarker development in CIRS, tracing its evolution from early functional tests such as visual contrast sensitivity (VCS) to advanced transcriptomic profiling. Drawing on peer-reviewed studies spanning two decades, we examine the layered integration of neuroendocrine, immunologic, metabolic, and genomic markers that collectively support a multisystem model of innate immune activation specific to environmentally acquired illness. Particular focus is given to the Gene Expression: Inflammation Explained (GENIE) platform’s use of transcriptomics to classify disease stages and distinguish CIRS from other fatiguing conditions. While ME/CFS research continues to explore overlapping pathophysiologic features, it has yet to establish a unified diagnostic model with validated biomarkers or exposure-linked mechanisms. As a result, many patients labeled with ME/CFS may, in fact, represent unrecognized CIRS cases. This review underscores the importance of structured biomarker timelines in improving differential diagnosis and guiding treatment in complex chronic illness and highlights the reproducibility of the CIRS framework in contrast to the diagnostic ambiguity surrounding ME/CFS.

## 1. Introduction

Chronic, unexplained multisystem illnesses such as Myalgic Encephalomyelitis/Chronic Fatigue Syndrome (ME/CFS) and fibromyalgia (FM) present a substantial diagnostic and therapeutic challenge [1]. A third, often overlooked condition—chronic inflammatory response syndrome (CIRS)—shares substantial symptom overlap with ME/CFS but features a reproducible pattern of immune dysregulation supported by biomarker data. Unlike allergic conditions, CIRS is not mediated by IgE or adaptive hypersensitivity, but instead reflects a dysregulated innate immune response to biotoxin or environmental exposure [2,3].

Affecting millions globally, ME/CFS is marked by disabling fatigue, post-exertional malaise (PEM), cognitive impairment, and autonomic symptoms, often leading to significant declines in function and quality of life [1,4,5]. Although the Centers for Disease Control and Prevention (CDC) and the National Academy of Medicine have issued formal case definitions—typically including PEM as a core feature—up to 90% of individuals with ME/CFS remain undiagnosed [4,6].

Despite decades of research, there remains no consensus on the pathogenesis of ME/CFS and no validated biomarker panel for diagnosis or treatment response [6,7,8]. Numerous studies have documented immune dysfunction, such as reduced natural killer (NK) cell cytotoxicity [9,10], immune checkpoint disruption [11], neuroinflammation [12], mitochondrial abnormalities [13], and altered T-lymphocyte (T cell) and NK cell metabolism [10]. Multi-omics and machine learning approaches have shown promise, identifying transcriptomic and microbial signatures in specific ME/CFS subtypes [14,15,16], but findings remain heterogeneous and lack clinical reproducibility [17,18]. Metabolomics and proteomics studies have also highlighted abnormalities in lipid metabolism, purine pathways, and complement activation, further supporting a systemic inflammatory process [13,19]. However, diagnostic precision remains elusive due to overlapping clinical presentations, varying exclusion criteria [7], and inconsistent diagnostic algorithms [6,20]. A brief summary table of these differences is provided in Table 1.

In contrast, CIRS—a progressive, under-recognized illness most frequently triggered by respiratory exposure to water-damaged buildings (WDBs), though other environmental exposures have been well documented—offers a model of reproducible innate immune dysregulation with a defined biomarker profile [21]. While often misdiagnosed as ME/CFS due to symptom overlap, CIRS is distinguishable by a panel of consistently altered markers, including elevated transforming growth factor beta 1 (TGF-β1), matrix metalloproteinase 9 (MMP-9), complement component 4a (C4a), and reduced melanocyte stimulation hormone, (MSH), as well as abnormalities in adrenocorticotropic hormone (ACTH)/cortisol, antidiuretic hormone (ADH)/osmolality, vascular endothelial growth factor (VEGF) and visual contrast sensitivity (VCS) [2]. Several case definitions for CIRS have been proposed and refined over the past two decades, incorporating symptom clusters, lab criteria, and documented response to treatment [22]. In contrast to the non-curative or marginally beneficial interventions for ME/CFS, the Shoemaker Protocol has demonstrated efficacy in treating CIRS, including objective biomarker normalization and symptom resolution in published studies [2].

This paper presents the biomarkers currently used in the clinical evaluation of CIRS, organized in the chronological order of their discovery, with a discussion of each biomarker’s physiological significance and diagnostic utility, and role in monitoring treatment response. Table 2 summarizes the key biomarkers and their development over time, providing a visual guide to the progression of CIRS biomarker identification. Tracing the historical development of the CIRS biomarker panel situates this condition within the broader framework of precision medicine for complex chronic illness. Given the substantial symptom overlap and lack of validated diagnostics in ME/CFS, many individuals with a current ME/CFS diagnosis may meet criteria for CIRS [2]. A structured evaluation of CIRS biomarkers offers clinicians a reproducible framework to rule in or rule out CIRS in patients presenting with ME/CFS-like illness, facilitating more accurate diagnosis and targeted intervention. By integrating biomarker-driven diagnostics into the evaluation of unexplained multisystem conditions, we propose a pathway toward more effective, individualized care.

## 2. Early Phase (1997–2004): The Neurotoxin Model and Visual Contrast Sensitivity

The origins of chronic inflammatory response syndrome (CIRS) trace back to clinical investigations into neurotoxic illness caused by estuarine exposure to Pfiesteria species in the mid-to-late 1990s. At that time, the illness was not yet understood as a systemic inflammatory condition but was instead conceptualized as a “neurotoxin-mediated illness,” based on observed deficits in cognition, memory, and contrast sensitivity in otherwise healthy individuals following environmental exposure. These early cases, referred to as Possible Estuarine-Associated Syndrome (PEAS), presented with symptoms such as memory loss, fatigue, diarrhea, and visual disturbances, including contrast sensitivity loss [23,39]. A pivotal early study by Grattan et al. [40] demonstrated that affected individuals exposed to Pfiesteria-contaminated waterways had significant impairments in verbal learning, divided attention, and fine motor coordination compared to controls, confirming the clinical neurotoxicity previously reported in Shoemaker’s patients.

A major innovation during this period was the introduction of visual contrast sensitivity (VCS) testing, developed by Dr. Ken Hudnell at the U.S. Environmental Protection Agency and adopted by Shoemaker as a screening and monitoring tool for PEAS. Shoemaker and Hudnell [23] confirmed that VCS deficits were common among exposed individuals and reliably responsive to treatment with cholestyramine (CSM), a bile acid sequestrant originally used to treat toxin-induced diarrhea. VCS provided a low-cost, reproducible, and quantifiable measure of visual pathway dysfunction, particularly at mid-to-high spatial frequencies, consistent with biotoxin-related neurotoxicity. In parallel, Shoemaker had begun empirically using CSM in patients with persistent diarrhea following environmental exposure and observed, unexpectedly, that it also improved neurocognitive symptoms and visual contrast disturbances [41]. These results suggested a broader toxin-binding mechanism, and VCS became the first objective marker in the evolution of the CIRS diagnostic framework.

At the same time, Shoemaker’s team began investigating Human Leukocyte Antigen (HLA) DR/DQ haplotypes, hypothesizing that genetic susceptibility might influence who became chronically ill following environmental exposure. By 2001, a formal HLA registry had been initiated to track allele distributions among patients with persistent illness after *Pfiesteria* or mold exposure [26]. Although no peer-reviewed stratification of haplotype-specific risk had yet been published, Shoemaker’s internal analyses by 2003 had already identified a disproportionate representation of specific genotypes across fungal, dinoflagellate, and tick-borne illness cohorts. This early use of HLA-based genetic susceptibility screening helped shift the conceptual model from toxic exposure to a host-response illness, wherein illness duration and severity were driven by an individual’s inability to adequately clear biotoxins [26]. By the early 2000s, the foundational components of CIRS—environmental exposure, genetic susceptibility, functional neurological impairment, and visual contrast disturbances—were coming into focus, though a formal case definition and biomarker panel had not yet appeared in the peer-reviewed literature.

## 3. Formative Phase (2005–2010): Multisystem Biomarker Validation and Structured Case Definitions

Between 2005 and 2010, the diagnostic model of CIRS transitioned from functional screening and clinical pattern recognition into a more rigorous, biomarker-driven framework. This shift was marked by the publication of the first formal case definition of chronic biotoxin-associated illness in 2005 [24]. In this chapter, Shoemaker and colleagues outlined a structured diagnostic approach based on a combination of symptom burden, objective laboratory abnormalities, and therapeutic response. Patients were required to meet exposure criteria, exhibit symptoms affecting four or more of eight physiological systems, and demonstrate abnormalities in at least three of six core biomarkers, including MSH, MMP-9, ACTH/cortisol, ADH/osmolality, VCS, and HLA DR/DQ. To confirm the diagnosis, patients also had to show improvement in at least two of three treatment-response indicators [24]. The publication marked a critical turning point, establishing CIRS as a reproducible illness with quantifiable diagnostic criteria.

A structured case definition for CIRS was supported by multiple study designs, including two randomized controlled trials, two case–control studies, several open-label and case series investigations, and multiple case reports. In total, the treatment literature from 2001 to 2017 includes two randomized controlled trials (*n* = 21), two case–control studies (*n* = 224 cases vs. 134 controls), five case-time series or open-label trials (*n* = 118), and seven case reports. Most of these studies evaluated therapeutic interventions such as cholestyramine or VIP and consistently demonstrated symptomatic improvement alongside normalization of inflammatory markers. These findings were instrumental in validating and applying the case definitions in clinical practice, showing that the diagnostic criteria reliably identified patients who responded to targeted treatment. A more detailed discussion of these studies appears in a recent review of CIRS treatment efficacy [2] and selected studies are also summarized in Table 2 of this manuscript.

Notably, in 2008, the U.S. Government Accountability Office (GAO), drawing on standards from HHS and the Institute of Medicine, described a general causation algorithm for environmentally linked illnesses that closely paralleled this diagnostic model. According to the GAO, demonstrating causation required three components: (1) epidemiologic association (i.e., evidence of exposure), (2) signs and symptoms consistent with the suspected illness, and (3) a reduction in symptoms following exposure removal or treatment [42]. Although the term CIRS had not yet been coined, the GAO’s retrospective alignment with Shoemaker’s framework lent additional support to its structured methodology. In essence, this framework served as a modern analog to Koch’s postulates for environmental illness—establishing causation through exposure, characteristic findings, and response to treatment—without requiring intentional re-exposure [43].

In parallel with the biomarker case definition, Shoemaker and colleagues developed a standardized screening tool composed of 13 symptom clusters to facilitate the early identification of CIRS. This symptom cluster model was later described in a 2017 study, where the presence of eight or more symptom clusters was reported to indicate a greater than 95% likelihood of CIRS-WDBs. When combined with VCS deficits, the authors stated that the diagnostic accuracy increased to 98.5% [3]. This symptom-based framework offered a non-invasive method for initial screening and became a widely used first-tier tool in clinical practice.

The same period saw the introduction or clinical validation of several key laboratory markers that remain central to the CIRS diagnostic panel today. Melanocyte-stimulating hormone (MSH) was found to be consistently low in patients with chronic exposure-related illness and was linked to dysregulation of downstream systems, including ACTH and ADH secretion [25,26]. MSH served as a keystone in the emerging understanding of hypothalamic dysfunction and loss of neuroimmune regulation, functioning as a master controller within the Shoemaker model [26]. Matrix metallopeptidase 9 (MMP-9), a marker of systemic inflammatory activation, was shown to be elevated in the majority of affected patients and to normalize with cholestyramine therapy [25,44]. The simultaneous measurement of ACTH and cortisol, and separately antidiuretic hormone (ADH) and plasma osmolality, provided insight into hypothalamic–pituitary dysfunction and volume regulation abnormalities. As summarized in Shoemaker & House [25], control participants demonstrated normal values for ACTH, cortisol, MSH, and ADH. By contrast, a majority of patients showed marked reductions in MSH and abnormalities in both endocrine axes.

Also notable during this phase was the evolving use of leptin as a marker of pro-inflammatory metabolic signaling. Elevated leptin levels were reported in over half of the patients in Shoemaker’s 2005–2006 studies and typically declined with treatment [25]. The consistent abnormalities seen across multiple, independently regulated systems—endocrine, immune, and neurologic—underscored the systemic nature of the illness and supported the model of a sustained innate immune response syndrome.

During this same period, initial associations between HLA DR/DQ haplotypes and illness following exposure to mold, dinoflagellates, or *Borrelia* were first reported by Shoemaker and Schmidt in 2005 [45], and subsequently expanded upon by Shoemaker in 2010. Shoemaker’s work built on registry data already being collected since 2001 and offered genotype-specific susceptibility profiles that were increasingly used to guide clinical risk stratification. Although no peer-reviewed stratification of haplotype-specific risk has yet been published, these early insights helped clarify why some individuals developed prolonged illness after minimal exposure, while others remained unaffected.

By the end of this formative phase, the diagnosis of CIRS had been anchored in both clinical symptomatology and a panel of reproducible laboratory findings, supported by published treatment protocols and response criteria. Although not yet widely adopted by conventional medicine, the model offered a structured, testable framework for identifying and treating patients with chronic illness following exposure to water-damaged buildings (WDB) and other biotoxin-containing environments.

## 4. Imaging and Neuroimmune Refinement (2010–2014)

Between 2010 and 2014, the CIRS model underwent further refinement as advances in brain imaging and immune profiling added depth to the diagnostic picture. This period marked a shift from static biomarker panels toward dynamic assessment of neuroimmune dysfunction, integrating structural and functional evidence of brain involvement. Shoemaker and colleagues reported reproducible patterns of brain volume changes in patients with biotoxin-associated illness using NeuroQuant^®^ (version 1.0; CorTechs Labs, Inc., San Diego, CA, USA), FDA-cleared volumetric MRI software. Statistically significant abnormalities were identified, including atrophy of the caudate nucleus and enlargement of the pallidum, left amygdala, and right forebrain parenchyma [28]. These patterns were absent in matched controls and were proposed as distinct neuroanatomical changes associated with CIRS.

The same study found a significant inverse correlation between forebrain volume and VEGF levels, supporting a mechanism involving inflammatory blood–brain barrier injury and capillary hypoperfusion. These volumetric changes were interpreted in the context of systemic immune dysregulation involving markers such as TGF-β1, MMP-9, VEGF, and C4a, which had previously been identified as elevated in affected patients [28].

During this period, the use of vasoactive intestinal polypeptide (VIP) expanded from diagnostic marker to therapeutic tool. In an open-label study of 20 patients who remained ill after completing the standard treatment protocol, VIP therapy produced significant clinical and immunological improvements [27]. Symptoms were reduced to control levels, and laboratory markers including C4a, TGF-β1, MMP-9, and VEGF normalized in most patients. Additionally, T regulatory cells (CD4+ CD25+) increased from a mean of 8.9% to 22.5%, indicating restoration of immunoregulatory function. The therapy was well-tolerated over 18 months, with no significant adverse events reported.

Further investigation into the CNS effects of VIP showed that nasal administration could reverse structural brain abnormalities in treatment-refractory patients. Although the study was published in 2017, it analyzed patients who had completed the full sequential protocol between 2010 and 2014, providing retrospective insight into outcomes from that period. Using serial MRI scans, the study demonstrated that higher-dose VIP therapy was associated with significant volumetric increases in grey matter nuclei, including the hippocampus, caudate, thalamus, putamen, pallidum, and cerebellum. In the cohort, the proportion of brain structures exhibiting overt atrophy fell below 1%, and nearly half of volumetric deficits were reversed. These improvements were not seen in control groups or in patients with enlarged lateral ventricles, reinforcing the therapeutic effect of VIP when used as the final step in the treatment protocol [33].

Taken together, these findings advanced the understanding of CIRS as a condition involving not only peripheral immune dysregulation but also central nervous system injury. The integration of volumetric imaging, neuroregulatory peptides, and inflammatory markers marked a turning point in the objective characterization of a systemic inflammatory illness involving neuroimmune, vascular, and endocrine pathways.

## 5. Transcriptomic Era Begins (2015–2017): From RNA-Seq to Targeted Expression Profiling

Between 2015 and 2017, the application of transcriptomic profiling transformed the diagnostic model of CIRS by providing a genome-wide view of immune and metabolic regulation. Initial discoveries from RNA sequencing (RNA-Seq) were translated into clinical diagnostics via the development of Gene Expression: Inflammation Explained (GENIE), a targeted transcriptomic platform based on Nanostring digital barcoding technology (Progene Dx, LLC, Bedford, Massachusetts) [21]. GENIE transcriptomic profiles are interpreted using Z-scores derived from a healthy control reference population [45], which anchors molecular abnormalities in a normative distribution and reduces the risk of circular reasoning during diagnostic evaluation.

Early transcriptomic investigations revealed broad suppression of genes encoding both large and small ribosomal subunits in untreated patients meeting CIRS criteria. These findings occurred alongside downregulation of nuclear-encoded mitochondrial genes and mitoribosomal genes, indicating a reproducible pattern of impaired protein synthesis and cellular energy production. This transcriptomic signature, termed molecular hypometabolism (MHM), emerged as a defining molecular hallmark of CIRS. As patients underwent treatment, these transcriptomic signatures shifted predictably, following a sequence of suppression, overshoot, correction, and eventual stability—a trajectory known as the CIRS curve [34].

In addition to MHM, transcriptomics revealed a second, complementary profile known as proliferative physiology, characterized by upregulation of IRS2 and downregulation of translocase. The result is a redirection of pyruvate from mitochondrial oxidative phosphorylation to cytoplasmic aerobic glycolysis, yielding lactic acid and promoting biosynthetic precursor production. This metabolic configuration produces far less adenosine triphosphate (ATP) than mitochondrial respiration and is associated with lactic acidosis, elevated anion gap, persistent fatigue, and impaired maximal oxygen consumption (VO_2_ max) [34]. Notably, IRS2 disruption may also result in intracellular insulin resistance through impaired glucose channel activation, independent of extracellular insulin signaling.

Transcriptomic analysis revealed interconnections between metabolic disruption and immune dysregulation. Patients with MHM and proliferative physiology commonly exhibit reduced T regulatory cell function, contributing to a chronic inflammatory state. Cytoskeletal injury, evidenced by upregulation of tubulin genes such as tubulin beta 1 class VI (*TUBB1*) and tubulin alpha 4a (*TUBA4A*), was also associated with closure of mitochondrial membrane pores and correlated with grey matter nuclear atrophy on imaging [46]. These gene expression changes helped explain the multisystem manifestations of CIRS, extending from cellular energetics to neuroimmune function.

Additional insight came from an analysis of the Ikaros family of transcription factors. Downregulation of *IKZF1* and vasoactive intestinal polypeptide receptor 1 (*VIPR1*) was associated with poorer clinical response to VIP and with increased grey matter atrophy, suggesting that transcriptional regulation plays a central role in both disease severity and therapeutic responsiveness. As transcriptomics matured, the GENIE platform enabled the dynamic assessment of these molecular profiles and their normalization over time, correlating with improvements in VCS, NeuroQuant metrics, and proteomic markers [30,46].

In 2015, Shoemaker and colleagues published a transcriptomic study of patients with chronic illness following exposure to ciguatera toxin—a marine biotoxin derived from dinoflagellate-contaminated fish. Using Affymetrix microarray analysis, the study identified a network of disrupted immune genes including Toll-interacting protein (*TOLLIP*), Single immunoglobulin and Toll-interleukin 1 receptor (TIR) domain) (*SIGIRR*), Vasoactive intestinal polypeptide receptor 2 (*VIPR2*), and Interleukin-18 receptor 1 (*IL18R1*), many of which overlapped with previously documented findings in CIRS-WDBs [29]. The authors proposed that chronic ciguatera illness represents a parallel form of CIRS, supporting the existence of a final common inflammatory pathway in response to diverse biotoxin triggers. This study reinforced the diagnostic utility of transcriptomics in distinguishing persistent immune dysregulation from other post-exposure syndromes.

In addition to patients exposed to water-damaged buildings or marine biotoxins, Shoemaker et al. [21] also applied GENIE profiling to individuals with a history of treated and untreated Lyme disease. Treated Lyme patients who met CIRS criteria demonstrated transcriptomic profiles with features overlapping other CIRS subtypes, including abnormal regulation of inflammation and persistent immune pathway disruption. These findings build on prior work by Bouquet et al. [47], who demonstrated that Lyme disease induces evolving transcriptomic changes across illness stages. In the acute, untreated phase, gene expression patterns reflected active infection and immune activation, with over 1200 genes differentially expressed. In contrast, patients six months post-treatment retained persistent abnormalities in over 600 genes, despite symptom resolution in some cases, supporting the concept of post-treatment Lyme disease as a chronic inflammatory response syndrome, or CIRS–Post-Lyme Syndrome (CIRS-PLS), with a transcriptomic profile distinct from acute infection.

Together, these data supported the model of Lyme-triggered CIRS as a post-infectious, immune-driven illness separable from active *Borrelia* infection by transcriptomic fingerprint. By the end of 2017, transcriptomics had become a central diagnostic and prognostic tool in the evaluation of CIRS. The integration of mitochondrial, ribosomal, glycolytic, immune regulatory, and cytoskeletal pathways facilitated a more complete molecular model of chronic cellular injury. In responders, VIP therapy completed the arc of transcriptomic normalization, demonstrating not only the specificity but the reversibility of the CIRS molecular signature [34].

## 6. Systems Integration (2017–2020): Diagnostic Convergence in Clinical Practice

Between 2017 and 2020, the diagnostic model for CIRS began to converge across systems. While the illness had long been understood as a multisystem, multi-symptom condition, this period marked a shift in how distinct biomarkers—imaging, proteomics, transcriptomics, and functional tests—were used together in clinical decision-making. Instead of isolated data points, these tools increasingly operated as integrated components of a unified diagnostic and treatment-monitoring framework. Although no single, large-scale validation study of the GENIE platform has been published to date, a growing body of transcriptomic studies in CIRS—spanning diagnostic classifiers, treatment response, and environmental correlations—has consistently shown alignment with established proteomic markers, symptom resolution, and neuroanatomical changes.

A major milestone in this convergence was the publication of the 2018 Consensus Statement, which outlined a standardized process for diagnosis based on a combination of symptom clusters, exposure history, VCS testing, HLA haplotypes, and a reproducible panel of laboratory biomarkers [21]. In addition to these core measures, the Consensus Statement incorporated advanced tools like NeuroQuant brain volumetrics and the GENIE transcriptomic platform, signaling a broadening of the diagnostic model to include neuroimaging and transcriptomic data alongside established biomarkers. The maturation of the GENIE tool played a central role in this evolution. By tracking expression patterns of ribosomal, mitochondrial, glycolytic, apoptotic, and regulatory genes, GENIE provided a dynamic window into cellular function. Crucially, these gene expression patterns could be correlated with findings on NeuroQuant—such as grey matter nuclear atrophy in the caudate, hippocampus, and thalamus—and with proteomic markers like MMP-9, VEGF, TGF-β1, and C4a [34,35,46]. This alignment enabled clinicians to correlate transcriptomic normalization (e.g., resolution of molecular hypometabolism) with measurable changes in brain structure, vascular tone, and symptom burden.

During this period, VIP therapy served both as a therapeutic intervention and a marker of transcriptomic recovery. In patients with transcriptomic profiles showing persistent suppression—particularly in ribosomal or mitochondrial genes—VIP administration was linked to gene expression normalization, decreased inflammatory markers, improved NeuroQuant volumes, and symptom resolution. This cross-system improvement provided one of the strongest examples of therapeutic integration, where transcriptomic, proteomic, and neuroanatomical recovery aligned [27,30,31,33].

A 2016 RNA-Seq study by Ryan and Shoemaker demonstrated that intranasal VIP therapy in 14 CIRS patients led to a significant reduction in symptom burden (mean dropped from 12.9 to 3.3) and improvement in proteomic markers such as TGF-β1 and C4a. Transcriptomic analysis showed coordinated downregulation of inflammatory and innate immune genes (e.g., granzymes, defensins, cluster of differentiation (CD) markers) along with suppressed ribosomal and mitochondrial gene expression, interpreted as a normalization of previously upregulated inflammatory pathways. Upregulation of Ikaros transcription factors further suggested restoration of immune regulation. These transcriptomic shifts coincided with clinical recovery and improvements on NeuroQuant imaging, supporting the role of transcriptomic normalization as a marker of therapeutic response in CIRS [30].

During this same period, McMahon [22] evaluated the applicability of alternate diagnostic strategies to broaden the clinical utility of CIRS identification. Recognizing that both the Shoemaker and GAO-derived case definitions required longitudinal treatment response to confirm diagnosis, McMahon analyzed the characteristics of 371 patients who met full diagnostic criteria. He found that these individuals consistently exhibited abnormalities in five or more of ten core biomarkers (or four in pediatric patients), or the failure of three validated screening tools.He then applied these statistically derived thresholds to the remaining 690 patients in his cohort, identifying an additional 302 likely CIRS cases. This resulted in a total of 673 patients who met either traditional or alternate diagnostic criteria—representing more than half of the cohort. These findings supported the use of pragmatic diagnostic adjuncts in early-stage illness and public health screening, where traditional case definitions may be impractical due to their reliance on treatment response.

Together, structured and adaptive diagnostic strategies converged during this period. While the interpretation of these multi-domain findings continued to rely on clinical judgment, the consistent correlation of results across modalities strengthened confidence in the model’s reproducibility. The use of GENIE, NeuroQuant, proteomic markers, and symptom tracking in tandem offered a multifaceted view of illness progression and response to therapy. As experience with these tools grew, patterns emerged that supported their integrated use in monitoring treatment success and guiding individualized care.

This period marked a turning point. For the first time, it became feasible to track illness progression and recovery using a modular, multi-system framework. Each tool—GENIE, NeuroQuant, proteomics, VCS—offered a distinct lens on the illness, and together, they formed a cohesive picture of CIRS as a reversible disorder of chronic immune and cellular injury.

## 7. Environmental Genomics and Causation (2021–22)

In 2021, the CIRS model advanced into a new phase with the application of transcriptomic tools to characterize not just the illness, but the environmental triggers that initiate and sustain it. This development marked a shift from association to causation. Where previous research had demonstrated consistent transcriptomic abnormalities in CIRS patients, the 2021 study by Shoemaker and colleagues was the first to demonstrate that gene expression profiles could differentiate among environmental exposures—supporting causality at the molecular level [35].

In a cohort of 50 patients with confirmed CIRS, GENIE transcriptomic analysis revealed distinct gene expression patterns that corresponded to specific types of environmental exposure. Patients with elevated expression of cluster of differentiation 14 (*CD14*) and toll-like receptor 4 (*TLR4*) were flagged as likely reactive to endotoxins. Those with increased transforming growth factor beta receptor 1 (*TGFBR1*) or transforming growth factor beta receptor 2 (*TGFBR2*) expression, often accompanied by activation of the mitogen-associated protein kinase (MAPK) pathway, were interpreted as consistent with exposure to ctinomycetes. The remaining patients displayed transcriptomic profiles suggestive of fungal or mycotoxin exposure, mast cell activation, or unresolved inflammatory responses to water-damaged buildings [35].

Importantly, several patients with actinomycetes-associated profiles had negative Environmental Relative Moldiness Index (ERMI) scores, demonstrating that non-fungal organisms—particularly biofilm-forming Actinobacteria—can trigger potent inflammatory responses that escape detection by conventional fungal DNA assays. Shoemaker and Lark [48] further proposed that mycolic acids, long-chain fatty acids found in Actinobacterial cell walls, could serve as candidate biomarkers for indoor Actinobacteria exposure, offering a potential analog to endotoxin testing for Gram-negative organisms. Shoemaker et al. [49] also reported that a significant correlation was found between Actinobacteria exposure, MAPK activation, and *TGFBR1/2* elevation in CIRS patients, supporting the mechanistic link between Actinobacteria exposure and transcriptomic inflammation, even in the absence of fungal growth on environmental sampling. This was further validated by the use of GENIE and transcriptomic assays which helped define the specific immune response related to Actinobacteria exposure [49].

These expression categories have since been used clinically to guide diagnostic reasoning. For example, patients with *TLR4/CD14* activation are often flagged for suspected endotoxin exposure, while those with MAPK and *TGFBR1* elevation are interpreted as consistent with inflammation associated with Actinobacteria exposure, including biofilm-forming species such as Corynebacterium, even when ERMI scores are negative—prompting alternative environmental investigation strategies [35].

This study formalized the dual-causation model in CIRS: (1) exposure to harmful environmental agents, and (2) genetically mediated host reactivity leading to persistent inflammation and cellular dysfunction. Together, these elements provide a structured basis for assessing environmentally acquired illness. The study’s findings offered both clinical and legal utility, advancing the field toward precision environmental genomics—a diagnostic approach that recognizes not only the presence of illness, but the transcriptomic fingerprint of the environmental source that caused it [35].

Later that year, Shoemaker and colleagues reported findings from a transcriptomic analysis of individuals with persistent symptoms following SARS-CoV-2 infection. In a comparison of 14 Post Covid Syndrome (PCS)-positive) and 7 PCS-negative individuals, the PCS-positive cohort exhibited molecular hypometabolism, proliferative physiology, and suppressed cluster of differentiation 3 delta chain (*CD3D*) expression—patterns previously observed in CIRS-WDBs. Transcriptomic signatures also revealed upregulation of *TGFBR1–3*, *CD14*, and *TLR4*, suggesting overlapping immune activation pathways. While these findings do not establish a causal mechanism, they raise the possibility that a subset of PCS patients may develop immune dysregulation consistent with the CIRS model. This extends the hypothesis that CIRS-like physiology can be triggered not only by environmental exposure but also by viral persistence or post-infectious immune injury [32].

In parallel, other research within this period focused on refining environmental exposure diagnostics. Building on mechanistic insights into actinobacterial pathogenesis, [48,49] EnviroBiomics, Inc. developed commercial tools such as *Actino Skin^®^* and *Actino Plasma^®^* to quantify Human Habitat (HH) Actinobacteria on the skin and evaluate host immune reactivity to mycolic acids. The *Actino Skin^®^* qPCR test detects species such as *Corynebacterium tuberculostearicum* and *Cutibacterium acnes* (formerly *Propionibacterium acnes*) and is currently associated with a pending U.S. patent application (No. 18/607,817). While formal validation of these tools is pending in the peer-reviewed literature, they reflect the expanding effort to translate molecular and environmental insights into actionable diagnostics.

## 8. Transcriptomic Expansion and Neuroimmune Risk Signaling (2023–2024)

From 2023 to 2024, the GENIE platform underwent critical expansion, enabling transcriptomic profiling to assess not only chronic inflammation and metabolic disruption, but also emerging signs of neurodegenerative risk. This new phase centered on the convergence of molecular markers associated with neuronal atrophy, cytoskeletal disruption, mitochondrial dysfunction, and vascular and coagulation pathways—revealing complex molecular trajectories in patients with persistent or late-stage CIRS.

In a 2023 study linking GENIE transcriptomics with brain volumetric MRI (NeuroQuant), Shoemaker and colleagues demonstrated that molecular hypometabolism (MHM) correlated with grey matter nuclear atrophy, cortical grey atrophy, and enlargement of the superior lateral ventricles. MHM, defined by suppressed ribosomal and mitochondrial gene expression, was strongly associated with exposure to water-damaged buildings, particularly to actinomycetes and endotoxins [46]. Importantly, these volumetric abnormalities improved following treatment with intranasal VIP, in parallel with transcriptomic correction [46].

Concurrently, GENIE data revealed a novel and concerning signature in a subset of CIRS patients: co-activation of *TUBB1*, *TUBA4A*, clusterin (*CLU*), and multiple coagulation-related genes (e.g., glycoprotein VI *(GP6)*, glycoprotein IX *(GP9)*, platelet factor 4 *(PF4)*, and integrin subunit alpha 2b (*ITGA2B*)), forming what was termed a “triple-positive” fingerprint. Initially observed in patients with Parkinson’s disease, this transcriptomic profile was later detected in younger, asymptomatic individuals with CIRS. Shoemaker et al. (2024) [38] proposed that this fingerprint may serve as an early risk indicator for neurodegeneration—potentially triggered or unmasked by environmental exposure—and may be modifiable through treatment. The presence of this fingerprint was associated with symptoms overlapping Parkinson’s—such as tremor, musculoskeletal pain, anosmia, and fatigue—and was shown to reverse with the Shoemaker Protocol and VIP in some cases. These findings raise the possibility that environmentally associated transcriptomic injury may contribute to or precede neurodegeneration. Further research is needed to determine whether this pattern represents a modifiable disease trajectory or a non-specific marker of neurodegenerative risk.

Further mechanistic insight came from Shoemaker et al., 2023 [36] linking MAPK pathway activation with upregulation of tubulin genes and elevated *CLU* in the same patients. These changes align with cytoskeletal collapse and oxidative injury patterns observed in other neurodegenerative processes, including Amyotrophic Lateral Sclerosis (ALS) and Alzheimer’s disease. While these findings do not redefine CIRS as a neurodegenerative disorder they highlight overlapping injury mechanisms that may converge in susceptible individuals. Prolonged immune activation, impaired mitochondrial energy production, coagulation-driven vascular compromise, and cytoskeletal destabilization may collectively increase neurodegenerative risk—particularly in genetically susceptible individuals exposed to biotoxins [36].

Building on this, recent transcriptomic research has identified upregulation of hypoxia-inducible factor 1-alpha (*HIF 1A*) in CIRS patients following exposure to water-damaged buildings. This molecular signature, typically associated with cellular hypoxia, reflects a shift toward proliferative physiology, characterized by impaired mitochondrial metabolism, increased glycolysis, and heightened inflammatory signaling. These abnormalities, tracked using the GENIE platform, were shown to normalize with VIP therapy, alongside improvements in surrogate markers of pulmonary hypertension and mitochondrial function [37].

By 2024, GENIE had evolved from a diagnostic confirmation tool into a stage-based platform for mapping molecular progression and treatment response in CIRS. Through the structured interpretation of gene expression patterns, patients could be stratified by transcriptomic phase—ranging from early molecular hypometabolism, to proliferative physiology, to partial recovery or neuroimmune risk profiles. These transcriptomic stages were interpreted in conjunction with clinical history, proteomic biomarkers, and environmental exposure data to inform causation, guide therapy, and monitor relapse risk. In this model, GENIE offered a reproducible molecular framework for assessing disease activity over time and individualizing treatment plans based on dynamic shifts in gene regulation.

## 9. Ongoing Validation and Future Research (2025–)

As of 2025, the diagnostic model of chronic inflammatory response syndrome (CIRS) continues to evolve through the ongoing validation of the GENIE transcriptomic platform and exploratory research into environmentally mediated neurodegenerative risk. GENIE remains central to clinical care, offering a dynamic, individualized profile of gene expression abnormalities that align with clinical stage, symptom burden, and treatment response [35]. Efforts are underway to standardize transcriptomic thresholds for staging recovery, monitoring relapse, and differentiating CIRS from other chronic inflammatory conditions [34].

Dr. Shoemaker’s current work is focused on refining these transcriptomic tools for the early detection of neuroimmune decline, particularly in individuals exhibiting “triple-positive” signatures associated with cytoskeletal breakdown, mitochondrial dysfunction, and pro-coagulant signaling [38]. This line of investigation seeks to clarify whether environmentally acquired immune injury may serve as a modifiable precursor to neurodegenerative disease, including conditions like Parkinson’s and ALS [38]. Preliminary evidence suggests that timely intervention with VIP and Shoemaker Protocol-based therapies may reverse early transcriptomic markers of degeneration, positioning CIRS treatment as both restorative and potentially preventive [34,38]. This ongoing validation reinforces the value of GENIE as both a diagnostic and prognostic tool and highlights the need for continued research to refine transcriptomic analysis, enhance staging precision, and identify patients at risk for persistent or progressive illness. Although GENIE has demonstrated alignment with clinical stage, treatment response, and biomarker normalization, further research is needed to evaluate the specificity of transcriptomic signatures within the broader landscape of chronic multisystem illness.

## 10. Discussion

Chronic, multisystem conditions such as Myalgic Encephalomyelitis/Chronic Fatigue Syndrome (ME/CFS) and chronic inflammatory response syndrome (CIRS) present overlapping clinical challenges but diverge in diagnostic precision and therapeutic reproducibility. ME/CFS remains a heterogeneous illness with elusive biomarkers, inconsistent case definitions, and limited treatment efficacy [2]. In contrast, CIRS is characterized by a structured case definition, reproducible biomarker panel, and measurable treatment response [2,24].

While ME/CFS interventions have historically centered on symptomatic management—including graded exercise therapy (GET), cognitive behavioral therapy (CBT), and off-label medications—these approaches have not been shown to normalize biomarkers or reverse disease pathophysiology. In contrast, the treatment of CIRS targets specific, reproducible abnormalities in immune and neuroendocrine markers, with the documented reversal of VCS deficits, reductions in inflammatory cytokines, normalization of transcriptomic profiles, and measurable symptom resolution. A comprehensive review of this evidence is provided in Dooley et al. [2]. This differential approach underscores the importance of accurate diagnosis and tailored therapeutic strategy.

This paper has traced the development of the CIRS diagnostic model from its functional roots in visual contrast sensitivity and HLA typing [24], through the integration of proteomics and volumetric neuroimaging, [28], to the recent expansion of transcriptomic platforms capable of identifying not only illness but its likely environmental trigger [35]. The GENIE platform now enables dynamic monitoring of molecular hypometabolism, immune dysregulation, and cytoskeletal injury—providing a personalized, molecular-level view of illness progression and treatment response [34].

Given the shared features of fatigue, cognitive impairment, post-exertional symptoms, and autonomic dysfunction, many individuals with a diagnosis of ME/CFS may in fact meet criteria for CIRS. Importantly, CIRS is a treatable condition. Although symptom onset in CIRS can be gradual—particularly in cases of chronic exposure to water-damaged buildings—it may also be abrupt, as seen in cases triggered by ciguatera toxin, Pfiesteria exposure, Lyme disease, or discrete environmental events such as remediation disturbance. The structured evaluation of biomarkers—including TGF-β1, MMP-9, C4a, MSH, and gene expression profiles—provides a reproducible diagnostic framework that can distinguish CIRS from other fatigue-related syndromes and guide intervention [3].

The evolution of the CIRS diagnostic framework illustrates how biomarker-guided precision medicine can reshape our understanding of complex, multisystem illnesses. While ME/CFS research continues to pursue consensus and reproducibility, CIRS represents a validated model of environmentally triggered immune injury—grounded in measurable pathophysiology, responsive to treatment, and aligned with principles of precision medicine [34,46]. Future research should consider whether subsets of ME/CFS patients might benefit from CIRS-based evaluation and intervention, and whether the structured, stage-based approach pioneered in CIRS could guide broader efforts to unravel multisystem, post-infectious, and environmentally acquired syndromes.

## Figures and Tables

**Table 1:** Comparative features of Chronic Inflammatory Response Syndrome (CIRS) and Myalgic Encephalomyelitis/Chronic Fatigue Syndrome (ME/CFS).

Feature	CIRS	ME/CFS
Pathogenesis	Innate immune dysregulation triggered primarily by environmental or biotoxin exposure	Unclear; proposed mitochondrial, immune, infectious, and neuroinflammatory roles
Diagnosis	Structured case definition using symptoms, biomarkers, and treatment response	Symptom-based case definitions; diagnosis of exclusion
Validated Biomarkers	Yes—defined and reproducible biomarker panel	No clinically validated biomarker panel
Reproducibility	High—reproducible patterns in diagnostic biomarkers and consistent treatment-response normalization across cohorts	Low—omics findings often not reproducible across cohorts
Treatment Response	Protocol-based; objective improvements in labs and symptoms documented	No curative treatment; management is supportive
Case Definition Origin	Biomarker-guided and treatment-responsive; refined over two decades	Multiple symptom-based definitions; heterogeneity persists
Symptom Overlap	Fatigue, cognitive dysfunction, post-exertional symptoms, autonomic dysregulation	Similar symptoms; post-exertional malaise (PEM) emphasized in recent definitions

**Table 2:** Chronological publication of biomarkers in CIRS ^1^.

Biomarker/Concept	Category	First Publication	Diagnostic Relevance	Type of Study
Visual contrast sensitivity (VCS)	Functional/Screening	*Environmental Health Perspectives*, 2001 [23]	Earliest objective test used in neurotoxic illness; confirmed reproducible in Possible Estuary-Associated Syndrome (PEAS)	Case series (5 patients)
Human leukocyte antigen DR/DQ (HLA DR/DQ) haplotypes	Genetic susceptibility	*Bioaerosols*, 2005 [24]	Used to determine risk for biotoxin illness across mold, Lyme, and dinoflagellate exposures	Case–control (156 cases/111 controls)
Melanocyte stimulating hormone (MSH)	Neuroendocrine	*Bioaerosols*, 2005 [24]	Key regulatory peptide; consistently low in CIRS patients	Case–control (156 cases/111 controls)
Matrix metalloproteinase 9 (MMP-9)	Inflammatory	*Bioaerosols*, 2005 [24]	Elevated in inflammatory response; used to monitor response to treatment	Case–control (156 cases/111 controls)
Multiple antibiotic-resistant coagulase-negative staphylococci (MARCoNS)	Infectious/Inflammatory	*Bioaerosols*, 2005 [24]	Associated with low MSH, persistent inflammation, and biofilm formation; eradication improves clinical and biomarker outcomes	Case–control (156 cases/111 controls)
Leptin	Metabolic	*Bioaerosols*, 2005 [24]	Elevated in CIRS; drops with therapy	Case–control (156 cases/111 controls)
Adrenocorticotropic hormone (ACTH)/Cortisol	Neuroendocrine	*Bioaerosols*, 2005 [24]	HPA axis dysregulation	Case–control (156 cases/111 controls)
antidiuretic hormone (ADH)/Osmolality	Hormonal/Fluid Balance	*Bioaerosols*, 2005 [24]	Volume dysregulation in MSH-deficient states	Case–control (156 cases/111 controls)
First formal case definition for Chronic Biotoxin Associated Illness (CBAI)	Diagnostic Framework	*Bioaerosols*, 2005 [24]	Exposure + symptoms + biomarkers + response	Case–control (156 cases/111 controls)
Vascular endothelial growth factor (VEGF)	Perfusion/Hypoxia	*Neurotoxicology and Teratology*, 2006 [25]	Biphasic; abnormal regulation indicates hypoxia, low capillary perfusion	Case time series (28 patients); randomized controlled trial (13 patients)
Complement component 4a C4a	Complement activation	*Surviving Mold*, 2010 [26]	Innate immune activation marker; rises with re-exposure	Clinical observation (uncontrolled; data compiled in *Surviving Mold*)
Vasoactive Intestinal Peptide (VIP)	Neuroendocrine	*Surviving Mold*, 2010 [26]	Key regulatory peptide	Clinical observation (uncontrolled; data compiled in *Surviving Mold*)
TGF-β1	Fibrosis/Cytokine	*Surviving Mold*, 2010 [26]	Pro-fibrotic cytokine; key inflammatory marker in CIRS	Clinical observation (uncontrolled; data compiled in *Surviving Mold*)
VIP	Neuropeptide/Therapeutic	*Health*, 2013 [27]	Restores immune regulation; corrects many CIRS abnormalities	Case–control (156 cases/111 controls)
NeuroQuant^®^ (Cortech Labs, Inc., San Diego, CA, USA) MRI	Neuroimaging	*Neurotoxicology and Teratology*, 2014 [28]	Quantifies grey matter changes (e.g., caudate atrophy); reversible with treatment	Case–control (17 cases/18 controls)
Caudate, hippocampus, thalamus, pallidum, putamen, cerebellum	Neuroimaging/Volumetric	*Neurotoxicology and Teratology*, 2014 [28]	NeuroQuant volumetric targets used to track structural brain changes associated with CIRS; reversible with VIP treatment	Case–control (17 case/18 controls)
Transcriptomic fingerprint (microarray)	Transcriptomics	*BMC Medical Genomics*, 2015 [29]	First transcriptomic classification of CIRS triggered by ciguatera toxin; clear gene pattern	Case–control (11 cases/11 controls)
RNA-Sequencing (RNA-Seq) post-VIP	Transcriptomics/Response	*Medical Research Archives*, 2016 [30]	Showed downregulation of ribosomal and mitochondrial genes after VIP	Prospective observational (14 cases)
*IKZF1* and *VIPR1*	Transcriptomic/Regulatory	*Medical Research Archives*, 2016 [30]	Downregulation linked to poor response to VIP and increased grey matter atrophy	Prospective observational (14 cases)
NeuroQuant reversal with treatment	Neuroimaging/Response	*Journal of Neuroscience & Clinical Research*, 2016 [31]	Grey matter and forebrain swelling reversed with treatment	Case–control (28 cases/23 controls)
Symptom clusters (13)	Clinical Screening	*Internal Medicine Review*, 2017 [32]	Diagnostic tool; ≥8/13 clusters predictive of CIRS	Retrospective observational analysis of clinical symptom data (*n* > 1000)
VIP-integrated imaging/lab study	Therapeutic Systems Integration	*Internal Medicine Review*, 2017 [33]	Showed lab normalization, grey matter volume restoration, and gene shift	Open-label trial (35 patients)
Translocase	Transcriptomic/Metabolic	*Trends in Diabetes and Metabolism*, 2020 [34]	Downregulated in proliferative physiology; contributes to altered pyruvate handling and reduced mitochondrial adenosine triphosphate (ATP) production	Cross-sectional cohort analysis (*n* = 112 consecutive Gene Expression: Inflammation Explained (GENIE) Stage 1 patients)
*IRS2*	Transcriptomic/Metabolic	*Trends in Diabetes and Metabolism*, 2020 [34]	Upregulated in proliferative physiology; indicates intracellular insulin resistance and altered glucose metabolism	Cross-sectional cohort analysis (*n* = 112 consecutive GENIE Stage 1 patients)
Molecular hypometabolism (MHM)	Transcriptomics/Diagnostic Classifier	*Trends in Diabetes and Metabolism*, 2020 [34]	Ribosomal and mitochondrial gene suppression; a core transcriptomic pattern in CIRS	Cross-sectional cohort analysis (*n* = 112 consecutive GENIE Stage 1 patients)
GENIE causation model	Causation/Transcriptomic Staging	*Medical Research Archives*, 2021 [35]	Defines stage-based diagnostic thresholds using GENIE + environmental exposure	Retrospective observational study with stage-stratified transcriptomic and environmental analysis (*n* ≈ 238 across Stages 0–5)
Actinobacteria	Environmental Trigger (NGS)	*Medical Research Archives*, 2021 [35]	First paper to link Actinobacterial presence in WDB environments with human transcriptomic signatures of inflammation (e.g., *MAPK1, TGFBR1*); supports gene-environment causation framework in CIRS	Retrospective observational study with stage-stratified transcriptomic and environmental analysis (*n* ≈ 238 across Stages 0–5)
Post-Covid Syndrome (PCS) vs. CIRS	Transcriptomic Comparison	*Medical Research Archives*, 2021 [32]	PCS patients with CIRS features showed MHM, *CD3D* suppression, *TGFBR* upregulation	Prospective observational study (*n* = 21 PCS patients; proof-of-concept transcriptomic analysis)
Actino Skin^®^ and Actino Plasma^®^	Translational/Diagnostic	Commercial product; patent pending ≈ 2022	Skin—quantitative polymerase chain reaction (qPCR)-based test for Human Habitat (HH)Actinobacteria on skin; supports exposure assessment in CIRS Plasma—quantifies immune reactivity to Actinobacterial mycolic acids; reflects systemic response in CIRS	Commercial assay development (patent pending; preliminary internal validation)
*TUBB1*, *TUBA4A*, Mitogen-associated protein kinase (MAPK)	Transcriptomic/Neurodegeneration	*Medical Research Archives*, 2023 [36]	Proposed markers for CNS injury (caudate atrophy) in CIRS	Retrospective observational study (386 of 1822 GENIE tests)
*HIF 1A*	Transcriptomic/Metabolic	*Medical Research Archives*, 2024 [37]	Upregulated following WDB exposure; reflects proliferative physiology marked by impaired mitochondrial metabolism, increased glycolysis, and heightened inflammatory signaling in CIRS	Retrospective observational study (81 of 1822 GENIE tests)
*CLU*, *GP6*, *GP9, PF4*, *ITGA2B*	Transcriptomic/Neuroimmune-Coagulation	*Medical Research Archives*, 2024 [38]	Co-expression of these genes defines the “triple-positive neuroimmune risk profile” in CIRS, associated with caudate atrophy, cytoskeletal disruption, and poor VIP response; overlaps with Parkinson’s disease transcriptomic signatures	Case series (77 patients); validation cohort (102 consecutive CIRS patients: 36 Triple Positives, 66 controls); retrospective analysis (171 of 1822 GENIE tests)

^1^ None of these biomarkers are exclusive to CIRS; many are altered in other inflammatory, infectious, or metabolic conditions. However, the specific constellation and co-occurrence of these biomarkers—alongside signs and symptoms, documented environmental exposure, and response to therapy—form the basis of the CIRS case definition described in Section 3. Some biomarkers were derived from peer-reviewed controlled studies, while others are based on unpublished clinical data collected by Dr. Shoemaker. Where available, sample size and use of control groups are noted.
